# ^89^Zr-Onartuzumab PET imaging of c-MET receptor dynamics

**DOI:** 10.1007/s00259-017-3672-x

**Published:** 2017-03-19

**Authors:** Martin Pool, Anton G. T. Terwisscha van Scheltinga, Arjan Kol, Danique Giesen, Elisabeth G. E. de Vries, Marjolijn N. Lub-de Hooge

**Affiliations:** 1 Department of Medical Oncology, University Medical Center Groningen, University of Groningen, Groningen, The Netherlands; 2 Department of Clinical Pharmacy and Pharmacology, University Medical Center Groningen, University of Groningen, P.O. Box 30.001, 9700 RB Groningen, The Netherlands; 3 Department of Nuclear Medicine and Molecular Imaging, University Medical Center Groningen, University of Groningen, Groningen, The Netherlands

**Keywords:** Onartuzumab, c-MET, HSP90, ^89^Zr, Erlotinib, PET

## Abstract

**Purpose:**

c-MET and its ligand hepatocyte growth factor are often dysregulated in human cancers. Dynamic changes in c-MET expression occur and might predict drug efficacy or emergence of resistance. Noninvasive visualization of c-MET dynamics could therefore potentially guide c-MET-directed therapies. We investigated the feasibility of ^89^Zr-labelled one-armed c-MET antibody onartuzumab PET for detecting relevant changes in c-MET levels induced by c-MET-mediated epidermal growth factor receptor (EGFR) tyrosine kinase inhibitor erlotinib resistance or heat shock protein-90 (HSP90) inhibitor NVP-AUY-922 treatment in human non-small-cell lung cancer (NSCLC) xenografts.

**Methods:**

In vitro membrane c-MET levels were determined by flow cytometry. HCC827ErlRes, an erlotinib-resistant clone with c-MET upregulation, was generated from the exon-19 EGFR-mutant human NSCLC cell line HCC827. Mice bearing HCC827 and HCC827ErlRes tumours in opposite flanks underwent ^89^Zr-onartuzumab PET scans. The HCC827-xenografted mice underwent ^89^Zr-onartuzumab PET scans before treatment and while receiving biweekly intraperitoneal injections of 100 mg/kg NVP-AUY-922 or vehicle. Ex vivo, tumour c-MET immunohistochemistry was correlated with the imaging results.

**Results:**

In vitro, membrane c-MET was upregulated in HCC827ErlRes tumours by 213 ± 44% in relation to the level in HCC827 tumours, while c-MET was downregulated by 69 ± 9% in HCC827 tumours following treatment with NVP-AUY-922. In vivo, ^89^Zr-onartuzumab uptake was 26% higher (*P* < 0.05) in erlotinib-resistant HCC827ErlRes than in HCC827 xenografts, while HCC827 tumour uptake was 33% lower (*P* < 0.001) following NVP-AUY-922 treatment.

**Conclusion:**

The results show that ^89^Zr-onartuzumab PET effectively discriminates relevant changes in c-MET levels and could potentially be used clinically to monitor c-MET status.

**Electronic supplementary material:**

The online version of this article (doi:10.1007/s00259-017-3672-x) contains supplementary material, which is available to authorized users.

## Introduction

c-MET is a receptor tyrosine kinase with roles in embryogenesis, and tissue repair and regeneration in homeostasis. Dysregulation of c-MET is often found in solid cancers as a consequence of mutation, protein overexpression, gene amplification and auto/paracrine upregulation of its ligand hepatocyte growth factor (HGF). Aberrant activation of c-MET/HGF signalling leads to increased tumour proliferation, invasiveness and angiogenesis, resulting in poor prognosis in various human cancers, including non-small-cell lung cancer (NSCLC) and gastric cancer [1]. NSCLC patients harbouring mutations activating epidermal growth factor receptor (EGFR) can be treated with the EGFR tyrosine kinase inhibitors (TKIs) erlotinib and gefitinib. Resistance of NSCLC to EGFR TKIs invariably occurs in these patients due to secondary mutations or activation of bypass signalling pathways, such as c-MET/HGF [2, 3]. Both c-MET and HGF have been implicated in resistance to EGFR-targeted therapies due to crosstalk via shared downstream signalling intermediates [2, 4]. Concurrent transient exposure to HGF can result in lasting resistance to EGFR targeting TKIs by providing positive selection pressure for c-MET-upregulated resistant subclones [5, 6]. c-MET-mediated resistance in EGFR TKI-resistant NSCLC was detected in around 5–15% of patients using quantitative polymerase chain reaction and fluorescence in situ hybridization [2, 7].

Several c-MET-directed treatment strategies have been reported. NVP-AUY-922, a non-geldanamycin heat shock protein 90 (HSP90) inhibitor, can potently downregulate EGFR and c-MET, and can overcome c-MET-mediated resistance in an EGFR TKI-resistant clone of NSCLC cell line HCC827 [8]. NVP-AUY-922 is currently in clinical trials in advanced EGFR mutant NSCLC refractory to EGFR TKIs (NCT01646125, NCT01124864), or with an EGFR exon 20 mutation (NCT01854034). The combination of NVP-AUY-922 and erlotinib in a phase I/II study of erlotinib-treated patients with progressive NSCLC has shown a 16% partial tumour response rate [9]. Another form of c-MET-directed therapy involves antibodies. Onartuzumab, is a 99-kDa one-armed antibody, reactive to human but not murine c-MET. Onartuzumab inhibits HGF binding, blocks c-MET receptor phosphorylation and downstream signalling and has shown antitumour efficacy in preclinical models [10].

 In a randomized phase II trial in patients with advanced NSCLC, the addition of onartuzumab to erlotinib resulted in an improvement in progression-free and overall survival in patients positive for c-MET on immunohistochemistry (IHC) [11]. However, the METLung randomized phase III clinical trial in which erlotinib was combined with onartuzumab or placebo in patients with NSCLC and positive for c-MET on IHC was terminated early because of lack of effectiveness [12]. MET amplification and overexpression, on the other hand, are associated with increased responses to c-MET inhibitors [13]. Recently, splice alterations in exon 14 of the MET gene (METex14), that are found in about 3% of human lung cancers, have been found to be associated with in vitro and clinical sensitivity to the c-MET TKI capmatinib and the ALK/ROS1/MET TKI crizotinib [14]. METex14 leads to impaired c-MET downregulation and degradation, resulting in c-MET protein overexpression [15].

Selection of patients with c-MET amplification or overexpression could therefore potentially be beneficial for therapy decisions, due to their sensitivity to c-MET-directed agents. IHC is generally performed on archived tissues and therefore reflects c-MET status of the tissues at the time of retrieval. c-MET status, however, can vary over time and among lesions, indicating a need for biomarkers able to capture this plasticity for the body as a whole [13, 16]. PET may provide a noninvasive method for assessing whole-body c-MET status to capture c-MET dynamics after the emergence of c-MET-mediated EGFR TKI resistance, or as response to HSP90 inhibition.

For ease of clinical translation, we selected the therapeutic antibody-based PET tracer ^89^Zr-onartuzumab, which has been reported to effectively discriminate c-MET expression in human gastric cancer xenografts [17]. Several other antibody and antibody fragment tracers have been reported to be of value for preclinical c-MET PET imaging [18]. These studies used an immunogenic full-length murine antibody DN30 [19] or antibody fragments and other protein scaffolds such as the ^89^Zr-labelled H2 cys-diabody, H2 minibody [20], and anticalin ^89^Zr-PRS110 [21]. In contrast to ^89^Zr-onartuzumab, these tracers are not readily translatable to the clinic, as none of these tracer protein backbones has yet been administered to patients, and would therefore require extensive safety testing. Furthermore, administration of tracers based on the HGF ligand such as ^64^Cu-HGF, as well as the bivalent antibody DN30 [19, 22], might have the unwanted result of stimulating tumour growth by activating c-MET.

We therefore aimed to validate the ability of ^89^Zr-onartuzumab PET to assess c-MET upregulation-mediated erlotinib resistance, as well as c-MET downregulation after HSP90 inhibitor NVP-AUY-922 treatment in human NSCLC xenografts.

## Materials and methods

### Cell lines and chemicals

The human NSCLC cell line HCC827 was obtained from the American Type Culture Collection. Cells were authenticated in April 2015 by STR profiling using BaseClear and quarantined until screening showed that they were negative for microbial and mycoplasma contamination. Cells were subcultured twice weekly using Roswell Park Memorial Institute-1640 (RPMI-1640) medium supplemented with 10% fetal calf serum (Bodinco BV) and incubated at 37 °C in a fully humidified atmosphere containing 5% CO_2_. HCC827ErlRes, a stable erlotinib-resistant subclone, was generated by culturing the parental cell line HCC827 in the presence of 1 μM erlotinib and 50 ng/mL recombinant human HGF (PeproTech) for 2 weeks, followed by 2 weeks in 1 μM erlotinib, a method analogous to that described by Turke et al. [6]. NVP-AUY-922 (Luminespib; LC Laboratories) and erlotinib (LC Laboratories) were dissolved in dimethyl sulfoxide, and aliquots stored at −80 °C.

### In vitro cell analyses

Surface expression of EGFR and c-MET was assessed using a BD Accuri™ C6 flow cytometer (BD Biosciences). Cetuximab (5 mg/mL; Merck) and onartuzumab (60 mg/mL; Genentech) served as primary antibodies for EGFR and c-MET, respectively, and mouse anti-human Fc-specific FITC-conjugated secondary antibody (clone HP-6017; Sigma-Aldrich) was used for readout of both EGFR and c-MET expression, with 10,000 events assessed per sample. The sensitivity of HCC827 and HCC827ErlRes cell lines to erlotinib and NVP-AUY-922 after 4 days of treatment was determined using the 3-(4,5-dimethylthiazol-2-yl)-2,5-diphenyltetrazolium bromide (MTT) assay [23].

### ^89^Zr-Onartuzumab tracer development and quality control

Onartuzumab was conjugated at five molar ratios (1:1, 1:2, 1:3, 1:5 and 1:10) of antibody to tetrafluorophenol-*N*-succinyldesferal-Fe^3+^ (Df; ABX GmbH), in triplicate for each ratio, as described previously [24]. Df-onartuzumab conjugates were checked for aggregation and fragmentation by size-exclusion high-performance liquid chromatography (SE-HPLC) using a Waters system equipped with a dual wavelength absorbance detector, in-line radioactivity detector and a TSK-GEL G3000SWXL column (JSB). Phosphate-buffered saline (PBS; 140 mmol/L NaCl, 9 mmol/L Na_2_HPO_4_, 1.3 mmol/L NaH_2_PO_4_; pH 7.4) was used as the mobile phase. The ratio of conjugated tetrafluorphenol-*N*-succinyldesferal-Fe^3+^ to antibody was determined by the antibody-bound versus unbound 430 nm signal of Fe^3+^ on SE-HPLC.

^89^Zr labelling was performed as described previously [25] using clinical grade ^89^Zr-oxalate (Perkin Elmer). Maximum attainable specific activity was determined by radiolabelling Df-onartuzumab conjugates with specific activities between 50 and 1,000 MBq ^89^Zr per milligram of conjugate. The radiochemical purity (RCP) of ^89^Zr-labelling was assessed by the trichloroacetic acid precipitation test [26]. The stability of ^89^Zr-onartuzumab was tested by incubation for 7 days under the following conditions: 0.9% NaCl at 4 °C, human serum at 37 °C and 0.5 M HEPES buffer, pH 7.2, at 37 °C.

The immunoreactivity of Df-onartuzumab conjugates was assessed by a competition assay as described previously. In brief, NuncBreakApart 96-well plates were coated overnight at 4 °C with 100 μL 100 ng/mL c-MET extracellular domain (Genentech) in 0.1 M Na_2_CO_3_ buffer, pH 9.6. ^89^Zr-Onartuzumab was diluted to 5,000 ng/mL in assay diluent which consisted of PBS, 0.5% bovine serum albumin fraction V (Bio-Connect) and 0.05% Tween 20 (Sigma-Aldrich). Plates were washed with washing buffer (PBS with 0.05% Tween 20) and blocked by incubation for 1 h at room temperature with 200 μL assay diluent, and washed again. Eight dilutions of unmodified onartuzumab at molar excesses between 0.004 and 156.25-fold were premixed 1:1 with diluted ^89^Zr-onartuzumab solution, and 100 μL aliquots of the mixtures were incubated in wells for 1 h at room temperature. After washing, the wells were broken apart and counted using a calibrated well-type 1282 Compugamma system (LKB Wallac). Counts were plotted against concentrations of competing unmodified onartuzumab and the half-maximum inhibitory concentration (IC50) was calculated using GraphPad 5.0 (GraphPad Software, Inc.). The IC50 was divided by the final tracer concentration (2,500 ng/mL) to yield the immunoreactive fraction.

### ^111^In-OA-NBC and ^89^Zr-OA-NBC control tracer

To determine nonspecific tracer distribution in organs, as well nonspecific tumour uptake, a one-armed isotype control antibody OA-NBC (Genentech) was coinjected and used as control tracer in all experiments. For ^111^In labelling, OA-NBC was conjugated with p-SCN-Bn-DTPA (Macrocyclics) as described previously [27]. Radiolabelling was performed with ^111^In-chloride (Mallinckrodt). The RCP of ^111^In-OA-NBC labelling was checked by silica gel instant thin-layer chromatography (ITLC) using 0.1 M citrate buffer, pH 6.0, as eluent. Furthermore, OA-NBC was incubated with a 1:5 molar excess of OA-NBC to Df, in a procedure similar to that described for Df-onartuzumab, to yield Df-OA-NBC conjugate for ^89^Zr-OA-NBC experiments.

### Animal experiments

Male nude mice (BALB/cOlaHsd-Foxn1^nu^; Envigo) were inoculated subcutaneously with xenograft tumours. Mice bearing only HCC827 xenograft tumours were injected subcutaneously with 5 × 10^6^ HCC827 cells into the right flank.

To determine the optimal tracer protein dose and imaging time-point, three groups each of four to six mice inoculated with HCC827 xenografts received 10, 25 or 100 μg ^89^Zr-onartuzumab (effective injected protein doses 9.7 ± 0.2, 24.0 ± 0.3 and 96.7 ± 0.9 μg at 4.24 ± 0.20, 4.57 ± 0.15 and 4.76 ± 0.13 MBq, respectively) coinjected with an equal amount of ^111^In-OA-NBC (1 MBq) isotype control via a penile vein. Tumour sizes were 183 ± 63, 234 ± 46 and 180 ± 92 mm^3^ in the groups treated with 10, 25 and 100 μg, respectively. All tracer injections contained 10 μg radiolabelled ^89^Zr-onartuzumab (specific activity about 400–500 MBq/mg) and 10 μg ^111^In-OA-NBC (specific activity about 100 MBq/mg), with cold onartuzumab and OA-NBC added to reach the total stated protein doses. Another group of three mice received 10 μg ^89^Zr-OA-NBC (effective protein doses 9.7 ± 0.3 μg at 4.47 ± 0.13 MBq) coinjected with 10 μg ^111^In-OA-NBC (1 MBq) to validate the use of ^111^In-OA-NBC as a proxy for the ^89^Zr-labelled control molecule. MicroPET scans were performed 1, 3 and 6 days after injection, followed by ex vivo biodistribution analysis after the final scan.

c-MET expression on HCC827ErlRes cells in comparison with that on HCC827 cells was evaluated by inoculating a group of ten mice with 5 × 10^6^ HCC827 cells into the right flank and 5 × 10^6^ HCC827ErlRes cells into the opposite flank. The sizes of the HCC827 and HCC827ErlRes tumours were 457 ± 182 and 240 ± 74 mm^3^ (*P* < 0.01), respectively, at the time of biodistribution analysis (day 6). Mice received 10 μg ^89^Zr-onartuzumab (effective protein dose injected 9.7 ± 0.2 μg at 4.42 ± 0.09 MBq) coinjected with 10 μg ^111^In-OA-NBC (1 MBq) via a penile vein. MicroPET scans were performed 6 days after injection, followed by ex vivo biodistribution analysis.

The effects of HSP90 inhibition on c-MET expression were evaluated in vivo using HCC827 xenograft-bearing mice. Mice bearing tumours of sizes 159 ± 65 and 208 ± 112 mm^3^ received 10 μg ^89^Zr-onartuzumab (effective protein dose injected 9.7 ± 0.1 and 9.8 ± 0.1 μg at 4.68 ± 0.15 and 4.44 ± 0.22 MBq for NVP-AUY-922 and vehicle, respectively) coinjected with 10 μg ^111^In-OA-NBC (1 MBq) via a penile vein on day 0. Scans were performed on day 6, after which the mice were treated by intraperitoneal injection with either 100 mg/kg NVP-AUY-922 in 5% glucose (seven mice) or 5% glucose vehicle (six mice) on days 6, 10, 13, 16 and 19. Mice received a second identical tracer injection on day 13 (effective protein dose injected 9.7 ± 0.1 and 9.6 ± 0.2 μg at 4.45 ± 0.36 and 4.41 ± 0.37 MBq for NVP-AUY-922 and vehicle, respectively), followed by microPET scans on day 19 and ex vivo biodistribution analysis. Tumours measured 205 ± 127 mm^3^in NVP-AUY-992-treated mice and 313 ± 198 mm^3^ in vehicle-treated mice at the time they were euthanised.

All microPET scans were performed using a Focus 220 rodent scanner (CTI Siemens). MicroPET scans were reconstructed and in vivo quantification was performed using AMIDE v. 1.0.4 [28]. Regions of interest (ROI) were drawn on the tumour based on the ex vivo weight assuming 1 g/cm^3^ tissue density or measured tumour volume for longitudinal experiments. Scan data are presented as mean standardized uptake values (SUV_mean_), which were calculated from the mean activity in the ROI divided by the injected dose (corrected for decay) per gram body weight, as described previously [29]. For biodistribution studies, organs and injected tracer standards were counted using the calibrated well-type LKB 1282 Compugamma system and weighed. ^111^In and ^89^Zr were counted in a single measurement at 311–500 keV and 758–1,144 keV, respectively, with crosstalk in the ^111^In channel corrected using a reference ^89^Zr dilution series. After decay correction, ex vivo tissue activities were expressed as the percentages of injected dose per gram tissue (%ID/g). Xenograft tumours were formalin-fixed and paraffin-embedded for IHC analysis. All animal experiments were approved by the Institutional Animal Care and Use Committee of the University of Groningen.

### Ex vivo analyses

Formalin fixed, paraffin-embedded tissue slices of thickness 4 μm were stained for c-MET using monoclonal rabbit anti-human c-MET antibody diluted 1:400 (ab51067; Abcam) [21]. Haematoxylin and eosin (H&E) staining was performed regularly to assess tissue viability and morphology. Digital scans of slides were acquired using a NanoZoomer 2.0-HT multislide scanner (Hamamatsu) and analysed with NanoZoomer Digital Pathology viewer software.

### Statistical analysis

Data are presented as means ± SD. Statistical analyses were performed with GraphPad Prism 5.0 using the two-sided Mann–Whitney test for nonparametric data and the two-sided paired Student’s *t* test for paired data. *P* values ≤0.05 were considered significant.

## Results

### Effects of erlotinib resistance and NVP-AUY-922 treatment on c-MET expression

**Fig. 1:**
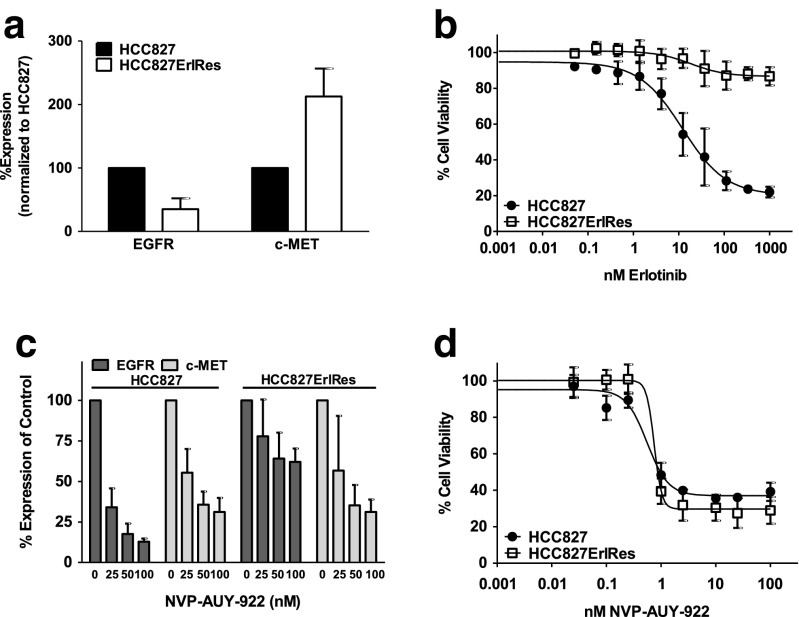
**a** In vitro flow cytometric analysis of EGFR and c-MET membrane expression in HCC827ErlRes cells normalized to expression in parental cell line HCC827. **b** In vitro MTT proliferation assay in HCC827 and HCC827ErlRes cells with exposure to increasing concentrations of erlotinib for 4 days. **c** In vitro flow cytometric analysis of EGFR and c-MET membrane expression in HCC827 and HCC827ErlRes cells after 24 h treatment with 25, 50 and 100 nM NVP-AUY-922 normalized to untreated controls. **d** In vitro MTT proliferation assay in HCC827 and HCC827ErlRes cells with exposure to increasing concentrations of NVP-AUY-922 for 4 days

An erlotinib-resistant clone, HCC827ErlRes, was generated from the parental cell line HCC827 by culturing cells for 2 weeks with 50 ng/mL HGF and 1 μM erlotinib, followed by 2 weeks culture in the presence of 1 μM erlotinib. Surface expression of c-MET on HCC827ErlRes cells as measured by flow cytometry was upregulated to 213 ± 44%, while EGFR surface levels were downregulated to 35 ± 17% of levels in the parental HCC827 cells (Fig. 1a). HCC827ErlRes cells were able to fully proliferate in the presence of up to 1,000 nM erlotinib as measured by the MTT assay, while parental HCC827 cells remained highly sensitive to erlotinib with an IC50 of 12 nM (Fig. 1b). NVP-AUY-922 treatment reduced surface expression of EGFR and c-MET (Fig. 1c). NVP-AUY-922 treatment was equally effective in reducing the viability of both HCC827 and HCC827ErlRes cells (Fig. 1d).

### ^89^Zr-onartuzumab tracer development

Conjugation of Df to onartuzumab was approximately 60% efficient for all molar reaction ratios tested (Supplementary Fig. 1a). Df-onartuzumab conjugates were able to consistently bind ≥500 MBq ^89^Zr per milligram of Df-onartuzumab with RCP ≥95% at ratios above 1:2 onartuzumab bound to Df (Supplementary Fig. 1b). The competition assay revealed a trend for lower immunoreactivity at higher conjugation ratios, signifying a need for balancing the required specific activity with the retained affinity of Df-onartuzumab conjugates (Supplementary Fig. 1c). Aggregation and fragmentation of Df-onartuzumab conjugates were not observed. A conjugation reaction ratio of 1:5, yielding 3.11 ± 0.33 Df bound to onartuzumab, was chosen as the optimal ratio for animal studies. ^89^Zr-Onartuzumab was stable in vitro, with a maximum observed decrease in RCP from 99.0 ± 0.2% to 91.0 ± 6.6% in human serum after 7 days at 37 °C (Supplementary Fig. 1d). All ^89^Zr-onartuzumab tracer batches had a RCP of ≥95% by trichloroacetic acid precipitation, while ^111^In-OA-NBC batches had a RCP of ≥90% by ITLC.

### ^89^Zr-Onartuzumab protein dose escalation

**Fig. 2:**
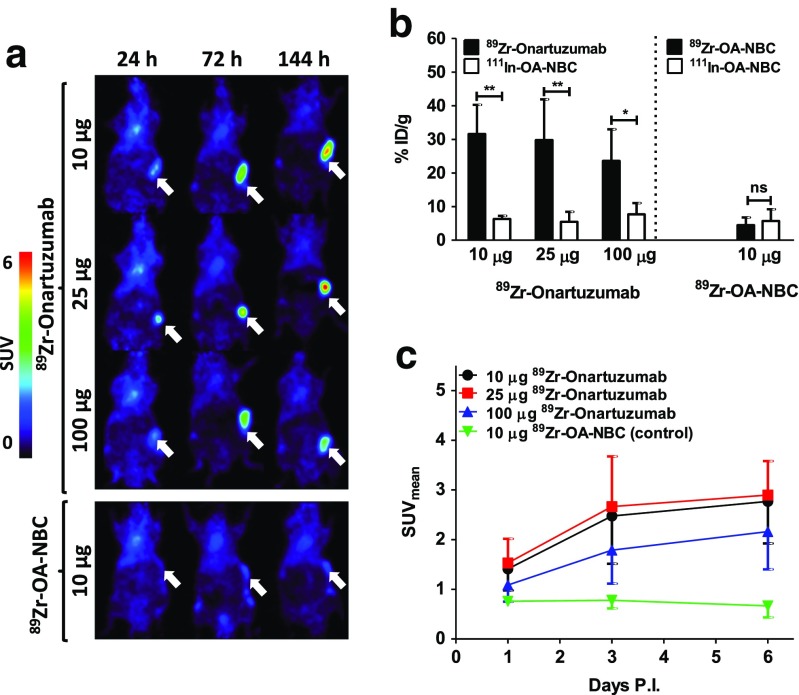
**a** Representative microPET scans of mice bearing HCC827 xenografts 24, 72 and 144 h after injection of 10, 25 and 100 μg ^89^Zr-onartuzumab (six, five and four mice, respectively) and 10 μg ^89^Zr-OA-NBC (three mice). **b** Ex vivo tumour uptake in HCC827 tumours 6 days after injection of 10, 25 and 100 μg ^89^Zr-onartuzumab and 10 μg ^89^Zr-OA-NBC compared with the ^111^In-OA-NBC control. **c** MicroPET quantification of uptake in HCC827 tumours 24, 72 and 144 h after injection of 10, 25 and 100 μg ^89^Zr-onartuzumab and 10 μg ^89^Zr-OA-NBC

^89^Zr-Onartuzumab tumour uptake increased over time for all tracer protein doses tested, with the highest tumour and least background organ uptake observed on day 6 after injection (Fig. 2a). ^89^Zr-Onartuzumab tumour uptake was higher than that of the coinjected ^111^In-OA-NBC control tracer in all tracer protein dose groups (Fig. 2b, Supplementary Fig. 2a, b). The 10 and 25 μg ^89^Zr-onartuzumab dose groups showed similar tumour uptakes of 31.6 ± 8.7 and 29.8 ± 12.1%ID/g, while uptake in the 100 μg ^89^Zr-onartuzumab dose group was lower but not significantly (*P* = 0.17 vs. the 10 μg group) at 23.5 ± 9.4%ID/g. SUV_mean_ values in tumours of the 10, 25 and 100 μg ^89^Zr-onartuzumab dose groups were 2.7 ± 0.9, 2.9 ± 0.7 and 2.2 ± 0.8, respectively (Fig. 2b, c, Supplementary Fig. 2a, b). The ^89^Zr-OA-NBC control showed no accumulation in HCC827 tumours over time (Fig. 2a, c, Supplementary Fig. 2a, b), while ex vivo tumour uptake was similar to that of ^111^In-OA-NBC (Fig. 2b, Supplementary Fig. 2a, b), showing the suitability of ^111^In-OA-NBC as a proxy for ^89^Zr-OA-NBC. Based on these results, a ^89^Zr-onartuzumab protein dose of 10 μg was used in the next mouse cohorts.

### In vivo effects of erlotinib resistance on ^89^Zr-onartuzumab PET

**Fig. 3:**
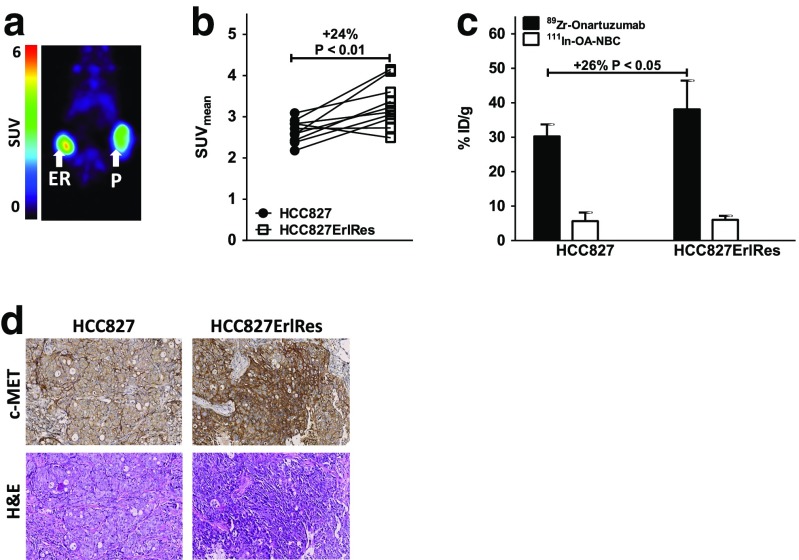
**a** Representative microPET scans in a representative mouse 144 h after injection of HCC827 cells (*P*, right flank) and HCC827ErlRes cells (*ER*, left flank). **b** MicroPET quantification of ^89^Zr-onartuzumab uptake in HCC827 and HCC827ErlRes tumours 144 h after injection of HCC827 and HCC827ErlRes cells. **c** Ex vivo uptake of ^89^Zr-onartuzumab and ^111^In-OA-NBC control in HCC827 and HCC827ErlRes tumours. **d** Ex vivo tissue analysis. c-MET and haematoxylin and eosin immunohistochemical staining in HCC827 and HCC827ErlRes tumours

PET scans showed 24% higher ^89^Zr-onartuzumab uptake in HCC827ErlRes tumours (SUV_mean_ 3.3 ± 0.5) than in HCC827 tumours (SUV_mean_ 2.7 ± 0.3; Fig. 3a, b; *P* < 0.01). Biodistribution analysis revealed that ^89^Zr-onartuzumab uptake was 26% higher in HCC827ErlRes tumours (38.1 ± 8.4%ID/g) than in HCC827 tumours (30.2 ± 3.5%ID/g; Fig. 3c, Supplementary Fig. 3, 6; *P* < 0.05). Similar comparisons of tumour-to-muscle and tumour-to-blood ratios for the two groups showed similar trends, but the differences did not reach significance. IHC revealed similar levels of necrosis, as well as higher c-MET expression in HCC827ErlRes than HCC827 tumours, in accordance with the ^89^Zr-onartuzumab PET and biodistribution data (Fig. 3d, Supplementary Fig. 5a).

### In vivo effects HSP90 inhibition on ^89^Zr-onartuzumab PET

**Fig. 4:**
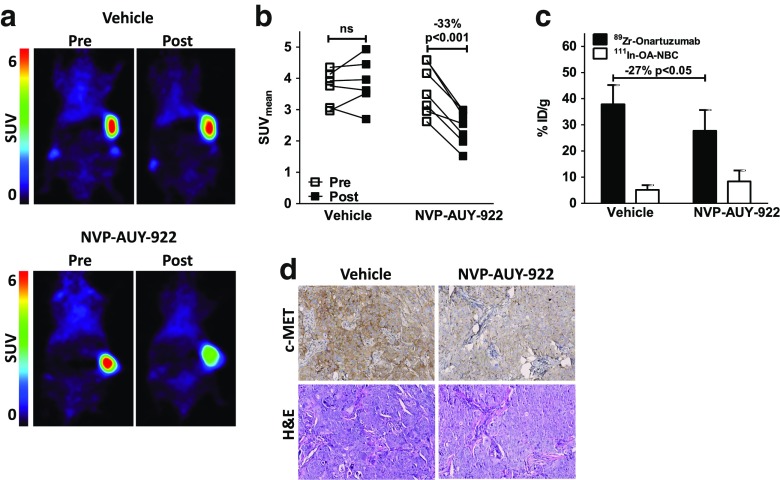
**a** Representative microPET scans in mice bearing HCC827 xenografts 144 h after injection, before treatment (day 6) and during treatment (day 19) with vehicle (six mice) or 100 mg/kg NVP-AUY-922 (seven mice). **b** MicroPET quantification of uptake in HCC827 tumours before treatment (day 6) and during treatment (day 19) with vehicle or 100 mg/kg NVP-AUY-922. **c** Ex vivo HCC827 tumour uptake (day 19) in vehicle-treated and NVP-AUY-922-treated mice (as %ID/g ^89^Zr-onartuzumab and ^111^In-OA-NBC control). **d** c-MET and haematoxylin and eosin immunohistochemical staining of tumours from vehicle-treated and NVP-AUY-922-treated mice

The effects of NVP-AUY-922 treatment on c-MET expression were evaluated in HCC827 xenograft-bearing mice. In mice receiving NVP-AUY-922, the tumour SUV_mean_ for ^89^Zr-onartuzumab was 33 ± 10% lower after treatment than in the baseline scans (*P* < 0.001), while uptake values in vehicle-treated mice before and during treatment were similar (Fig. 4a, b). Biodistribution studies confirmed the PET results revealing 27% lower ^89^Zr-onartuzumab tumour uptake in NVP-AUY-922-treated mice than in vehicle-treated mice (Fig. 4c, Supplementary Figs. 4a, 4b, 6; *P* < 0.05). Similar comparisons of the tumour-to-muscle and tumour-to-blood ratios for the two groups showed similar trends, but the differences did not reach significance. IHC showed a marked decrease in c-MET expression in the tumours, with similar levels of necrosis, in NVP-AUY-922-treated and in vehicle-treated animals (Fig. 4d, Supplementary Fig. 5b).

## Discussion

This study illustrates the feasibility of in vivo imaging of c-MET dynamics by detecting upregulation after c-MET-mediated erlotinib resistance, as well as downregulation of c-MET following HSP90-directed therapy using ^89^Zr-onartuzumab PET in human NSCLC xenograft-bearing mice. Monitoring c-MET in vivo with ^89^Zr-onartuzumab PET might be an attractive method for detecting c-MET-mediated emergence of resistance to EGFR TKIs, as well as for assessing downregulation of c-MET in response to HSP90 inhibition. Furthermore, it could be beneficial for patient selection for c-MET-directed therapy, as MET amplification and overexpression are associated with increased response to c-MET inhibitors [13].

Because of the different Df-suc-*N*-onartuzumab linker chemistry used for ^89^Zr radiolabelling in the present study and for ^89^Zr-Df-Bz-SCN-onartuzumab in the study by Jagoda et al. [17], we performed a tracer protein dose escalation study to optimize the ^89^Zr-onartuzumab protein dose and imaging time-point. HCC827 tumours in the present study showed high contrast and c-MET-specific ^89^Zr-onartuzumab uptake at a tracer protein dose of 10 μg. Higher ^89^Zr-onartuzumab protein doses showed a trend for lower tumour uptake. This blocking effect was also shown by Jagoda et al. using 1 mg cold onartuzumab, and is most likely caused by its nonreactivity with murine c-MET [10, 17]. ^89^Zr-Onartuzumab is remarkable in that respect, in that specific binding can be proven in vivo in a conventional blocking study. In contrast, cross-reactive antibodies might need a higher protein dose for optimal tumour contrast [30]. Similar protein doses of ^89^Zr-OA-NBC and ^111^In-OA-NBC one-armed isotype controls for ^89^Zr-onartuzumab did not accumulate in tumour tissues, confirming the c-MET specificity of the ^89^Zr-onartuzumab signal. ^89^Zr-Df-p-SCN-onartuzumab uptake in MKN-45 gastric cancer xenografts 5 days after injection was lower at 22.5%ID/g than observed for ^89^Zr-Df-suc-*N*-onartuzumab in HCC827 xenografts [17]. A direct comparison, however, is difficult due to differences in tumour models, desferal linker chemistries, tracer protein doses and day of biodistribution analysis.

Upregulation of c-MET in EGFR TKI gefitinib-resistant HCC827-GR6 xenografts was visualized with a ^89^Zr-labelled H2 cys-diabody and H2 minibody, with twofold higher uptake observed in resistant tumours [20]. The reported biodistribution data for the resistant tumours, however, excluded cystic areas, which affected approximately half of the resistant c-MET-upregulated HCC827-GR6 tumours. Exclusion of these cystic areas might have artificially increased the apparent uptake of these tracers. Furthermore, a highly variable absolute tumour uptake was observed, differing by up to twofold between experiments, correlating with a similar variation in the remaining blood pool activity [20]. We observed robust tumour uptake of ^89^Zr-onartuzumab. As the affinity of the H2 cys-diabody and H2 minibody for c-MET are comparable to that of onartuzumab, this is probably due to the high molecular weight of ^89^Zr-onartuzumab that results in slower blood clearance and prolonged tumour exposure [10, 20]. ^89^Zr-Onartuzumab tumour-to-muscle and tumour-to-blood ratios showed similar trends, but the differences between HCC827 and HCC827Erlres tumours did not reach significance. However, we included the ^111^In-OA-NBC paired control molecule in each experimental animal as a valid control to determine specific tumour uptake [31], as well as ex vivo analysis for c-MET expression and necrosis. This strengthens the conclusion that the extra uptake in c-MET-upregulated HCC827ErlRes xenografts is indeed mediated by c-MET expression, and is not caused by possible variations in tumour size, blood pool tracer availability and permeability effects.

Pharmacokinetic readout of c-MET in response to c-MET TKI PHA665752 therapy has shown reduced total MET protein in human gastric cancer MKN-45 xenografts due to more necrosis which coincides with lower uptake of c-MET-specific peptide ^99M^Tc-AH-113018 [32]. The AH113804 peptide has also been labelled with ^18^F for PET, and is able to detect recurrence of c-MET-expressing basal-like breast cancer xenografts after surgical resection [33]. A fluorescent labelled version of the same peptide has been used as a ‘red flag’ technique to detect polyps using a fluorescence endoscope technique in patients at risk of developing colon cancer [34].

We and others have shown potent downregulation of both EGFR and c-MET in human NSCLC cell lines by exposure to HSP90 inhibitors NVP-AUY-922 and 17-DMAG [8, 35]. c-MET downregulation by treatment with NVP-AUY-922 has also been shown to overcome c-MET-mediated gefitinib and erlotinib acquired resistance in HCC827 sub clones [8]. We have visualized this downregulation of c-MET by treatment with NVP-AUY-922 in vivo using ^89^Zr-onartuzumab PET, with sequential scans showing 33% lower uptake after treatment. This correlated with downregulation of c-MET protein on ex vivo IHC, while tumour necrosis levels were similar in NVP-AUY-922-treated and vehicle-treated animals.

A limiting factor in the present study may have been the relatively small effect sizes observed for upregulation and downregulation of tracer tumour accumulation. Preclinical studies using ^89^Zr-labelled antibody tracers have shown that HSP90 inhibition and everolimus treatment downregulate other key oncogenic proteins, such as HER2, vascular endothelial growth factor A (VEGF-A), and insulin-like growth factor 1 receptor (IGF1R). The effect sizes of antibody tracer uptake reduction after target protein downregulation through HSP90 inhibition and everolimus treatment are comparable to the reduction in uptake of ^89^Zr-onartuzumab after c-MET downregulation found in the present study [36–39], and the results of some of these preclinical studies have been translated to successful treatments in the clinic [40, 41]. Furthermore, insight into whole-body c-MET target distribution via noninvasive ^89^Zr-onartuzumab scans could potentially enlarge the patient population which might benefit from c-MET/HGF-targeted drugs [18].

Based on these promising preclinical results, we conclude that ^89^Zr-onartuzumab c-MET PET provides a robust noninvasive and powerful tool that is easily translatable to the clinic for visualizing c-MET dynamics as a possible biomarker for c-MET-mediated resistance to erlotinib and for treatment response to HSP90 inhibition.

## Electronic supplementary material

Below is the link to the electronic supplementary material.

Supplementary Fig. 1 **a** Efficiency of conjugation of Df to onartuzumab at various molar reaction ratios. **b** Radiochemical purity for ^89^Zr labelling at various Df to onartuzumab molar conjugation ratios, with specific activities between 50 and 1,000 MBq/mg conjugate. **c** Retained affinity of ^89^Zr-onartuzumab at various Df to onartuzumab molar conjugation ratios compared with that of naked onartuzumab in a competition assay. **d** Stability of ^89^Zr-onartuzumab in 0.9% NaCl at 4 °C, human serum at 37 °C and HEPES, pH 7.2 at 37° °C up to 7 days (PDF 38 kb)

Supplementary Fig. 2 **a** Ex vivo organ uptake in HCC827 xenograft-bearing mice of ^89^Zr-onartuzumab 6 days after injection at protein doses of 10, 25 and 100 μg (six, five and four mice, respectively) and uptake of ^89^Zr-OA-NBC 6 days after injection at a protein dose of 10 μg. **b** Corresponding ex vivo organ uptake in HCC827 xenograft-bearing mice of ^111^In-OA-NBC 6 days after injection. Data are expressed as %ID/g ± SD (PDF 48 kb)

Supplementary Fig. 3 Ex vivo organ uptake of ^89^Zr-onartuzumab and ^111^In-OA-NBC both 6 days after injection at a dose of 10 μg in ten HCC827 and HCC827ErlRes tumour-bearing mice. Data are expressed as %ID/g ± SD (PDF 35 kb)

Supplementary Fig. 4 **a** Ex vivo organ uptake of ^89^Zr-onartuzumab 6 days after injection in six vehicle-treated and seven NVP-AUY-922-treated (at a dose of 100 mg/kg) HCC827 xenograft-bearing mice. **b** Corresponding ex vivo organ uptake of ^111^In-OA-NBC 6 days after injection in HCC827 xenograft-bearing mice. Data are expressed as %ID/g ± SD (PDF 42 kb)

Supplementary Fig. 5 Histological grading of necrosis (H&E staining) of (**a**) HCC827 and HCC827ErlRes tumours and (**b**) vehicle-treated and NVP-AUY-922-treated HCC827 tumours, where score 0+ represents 0–5% necrosis, 1+ 5–15% necrosis, 2+ 15–25% necrosis, 3+ 25–35% necrosis and 4+ >35% necrosis. (PDF 25 kb)

Supplementary Fig. 6 Correlation between ex vivo ^89^Zr-onartuzumab/^89^Zr-OACD8 tumour uptake (%ID/g) and in vivo PET tumour uptake (SUV_mean_). (PDF 31 kb)
