# Supplementary material for: 89Zr-Onartuzumab PET imaging of c-MET receptor dynamics
Source: Eur J Nucl Med Mol Imaging. 2017 Mar 19;44(8):1328–36. doi: 10.1007/s00259-017-3672-x (PMC5486818; doi:10.1007/s00259-017-3672-x)

Supplementary Fig. 1 (a) Conjugation reaction efficiency of Df to onartuzumab at varying molar reaction ratios. **(b)** Radiochemical purity for ^{89}Zr labelling at various conjugation ratios for Df to onartuzumab, with specific activities between 50-1000 MBq/mg conjugate. **(c)** Retained affinity of ^{89}Zr-onartuzumab at varying molar conjugation ratios of Df to onartuzumab, compared to naked onartuzumab by competition assay. **(d)** Stability of ^{89}Zr-onartuzumab in 0.9% NaCl at 4° C, human serum at 37° C and HEPES pH 7.2 at 37° C up to 7 days

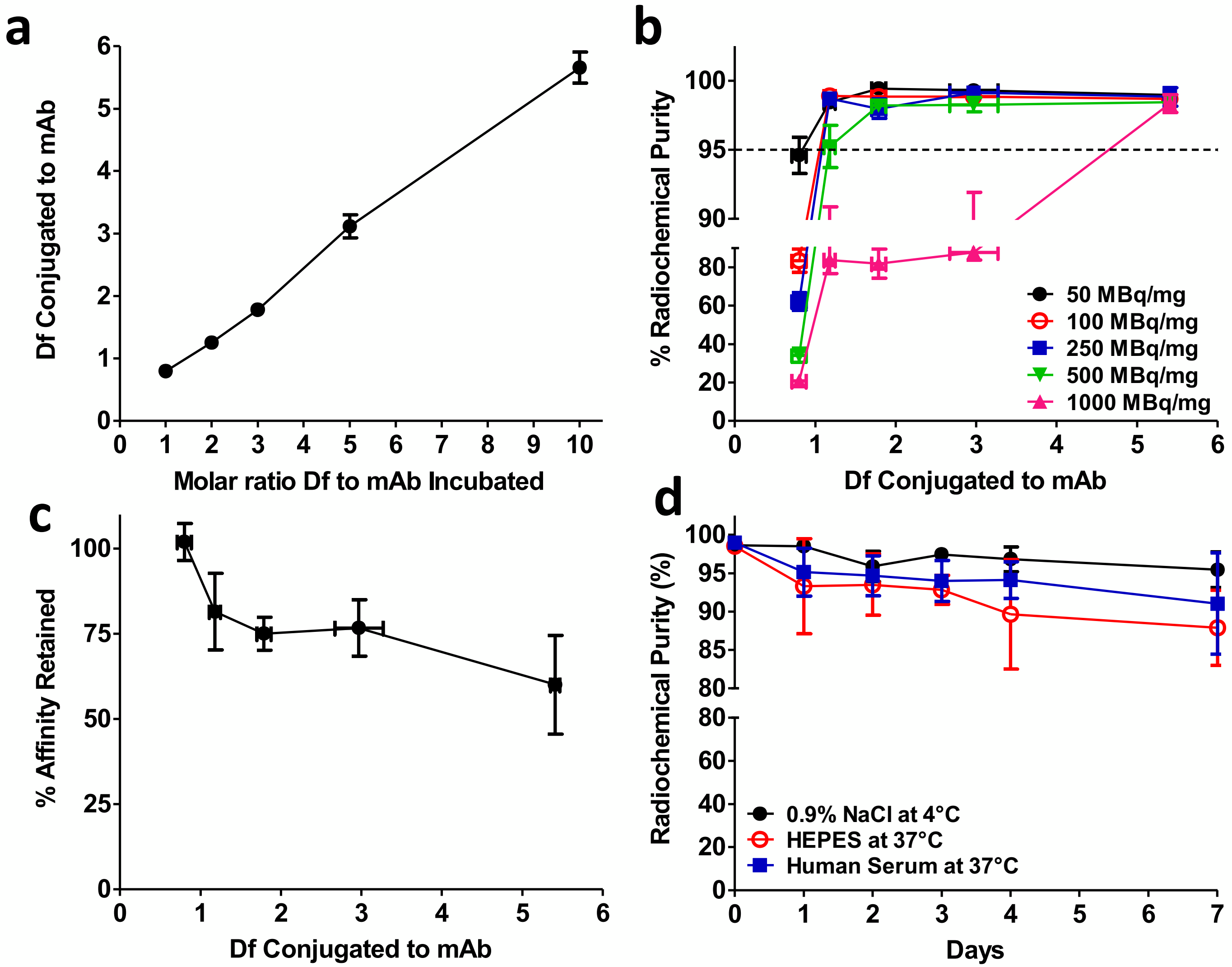

Supplement: Supplementary file 1 — a Efficiency of conjugation of Df to onartuzumab at various molar reaction ratios. b Radiochemical purity for 89Zr labelling at various Df to onartuzumab molar conjugation ratios, with specific activities between 50 and 1,000 MBq/mg conjugate. c Retained affinity of 89Zr-onartuzumab at various Df to onartuzumab molar conjugation ratios compared with that of naked onartuzumab in a competition assay. d Stability of 89Zr-onartuzumab in 0.9% NaCl at 4 °C, human serum at 37 °C and HEPES, pH 7.2 at 37° °C up to 7 days (PDF 38 kb) [file 259_2017_3672_MOESM1_ESM.pdf]
