# Supplementary material for: 89Zr-Onartuzumab PET imaging of c-MET receptor dynamics
Source: Eur J Nucl Med Mol Imaging. 2017 Mar 19;44(8):1328–36. doi: 10.1007/s00259-017-3672-x (PMC5486818; doi:10.1007/s00259-017-3672-x)

Supplementary Fig. 2 (a) *Ex vivo* organ uptake of 10, 25 and 100 µg ^{89}Zr-onartuzumab and 10 µg ^{89}Zr-OA-NBC tracer protein doses, 6 days pi and **(b)** corresponding *ex vivo* organ uptake ^{111}In-OA-NBC in HCC827 xenograft bearing mice, 6 days pi. Data are expressed as % ID/g ± SD

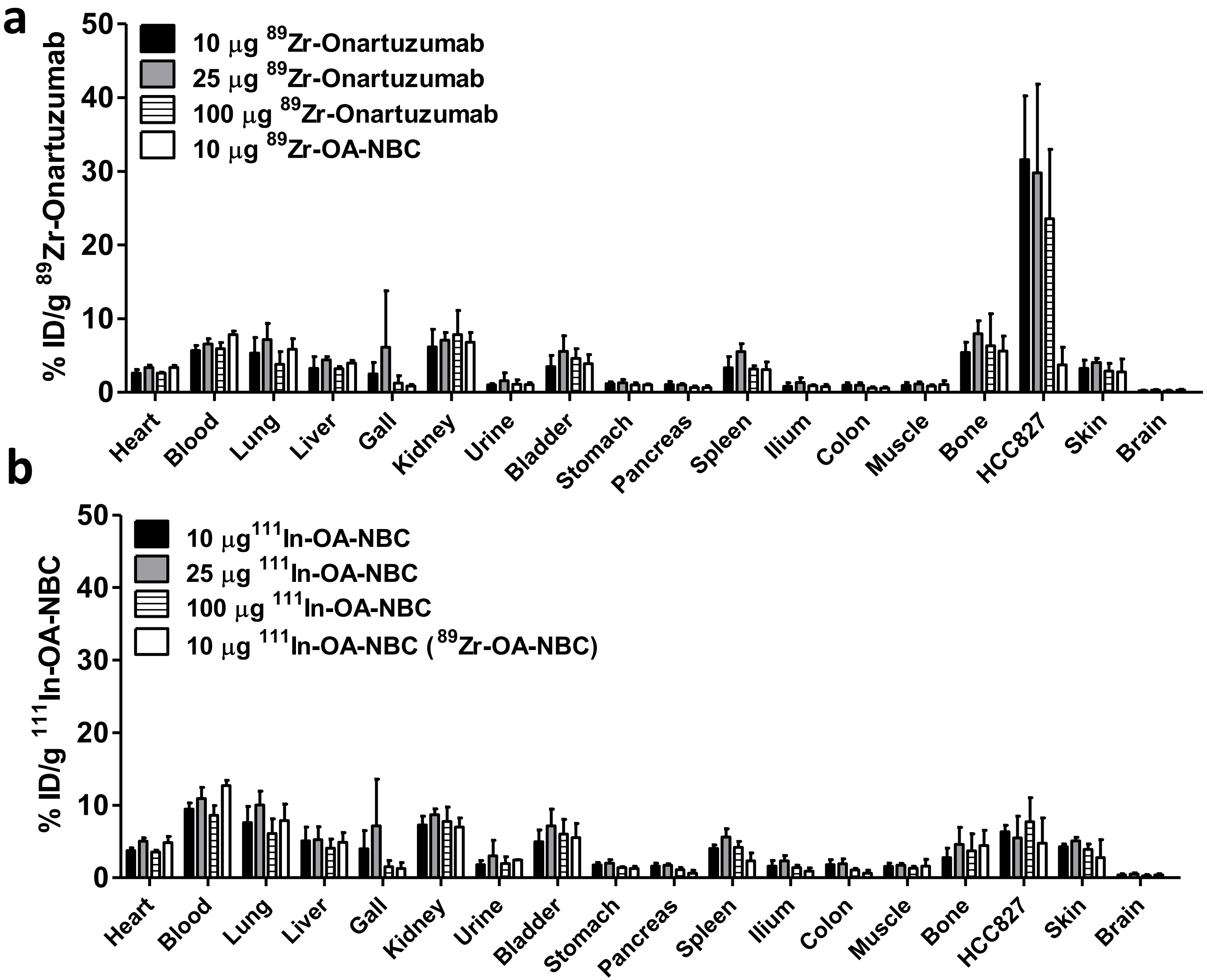

Supplement: Supplementary file 2 — a Ex vivo organ uptake in HCC827 xenograft-bearing mice of 89Zr-onartuzumab 6 days after injection at protein doses of 10, 25 and 100 μg (six, five and four mice, respectively) and uptake of 89Zr-OA-NBC 6 days after injection at a protein dose of 10 μg. b Corresponding ex vivo organ uptake in HCC827 xenograft-bearing mice of 111In-OA-NBC 6 days after injection. Data are expressed as %ID/g ± SD (PDF 48 kb) [file 259_2017_3672_MOESM2_ESM.pdf]
