# Supplementary material for: 89Zr-Onartuzumab PET imaging of c-MET receptor dynamics
Source: Eur J Nucl Med Mol Imaging. 2017 Mar 19;44(8):1328–36. doi: 10.1007/s00259-017-3672-x (PMC5486818; doi:10.1007/s00259-017-3672-x)

Supplementary Fig. 3 *Ex vivo* organ uptake of 10 µg ^{89}Zr-onartuzumab and 10 µg ^{111}In-OA-NBC in HCC827 and HCC827ErlRes tumour bearing mice, 6 days pi. Data are expressed as % ID/g ± SD

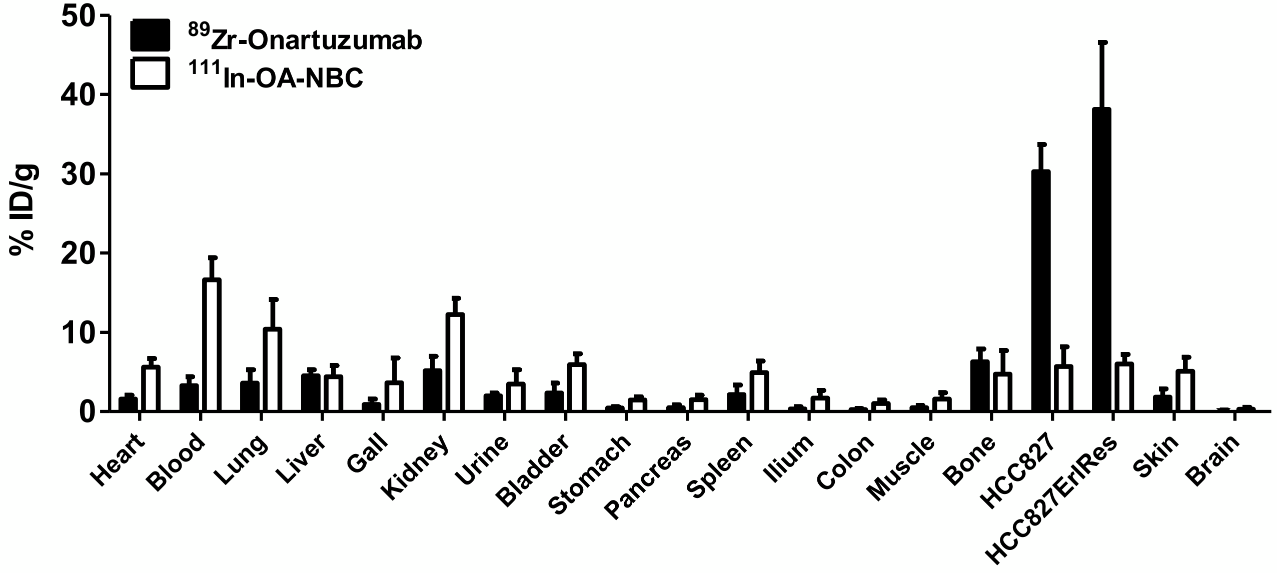

Supplement: Supplementary file 3 — Ex vivo organ uptake of 89Zr-onartuzumab and 111In-OA-NBC both 6 days after injection at a dose of 10 μg in ten HCC827 and HCC827ErlRes tumour-bearing mice. Data are expressed as %ID/g ± SD (PDF 35 kb) [file 259_2017_3672_MOESM3_ESM.pdf]
