# Supplementary material for: 89Zr-Onartuzumab PET imaging of c-MET receptor dynamics
Source: Eur J Nucl Med Mol Imaging. 2017 Mar 19;44(8):1328–36. doi: 10.1007/s00259-017-3672-x (PMC5486818; doi:10.1007/s00259-017-3672-x)

Supplementary Fig. 4 (a) *Ex vivo* organ uptake of 10 µg ^{89}Zr-onartuzumab in vehicle and 100 mg/kg NVP-AUY-922 treated mice, 6 days pi and **(b)** corresponding *ex vivo* organ uptake ^{111}In-OA-NBC in HCC827 xenograft bearing mice, 6 days pi. Data are expressed as % ID/g ± SD

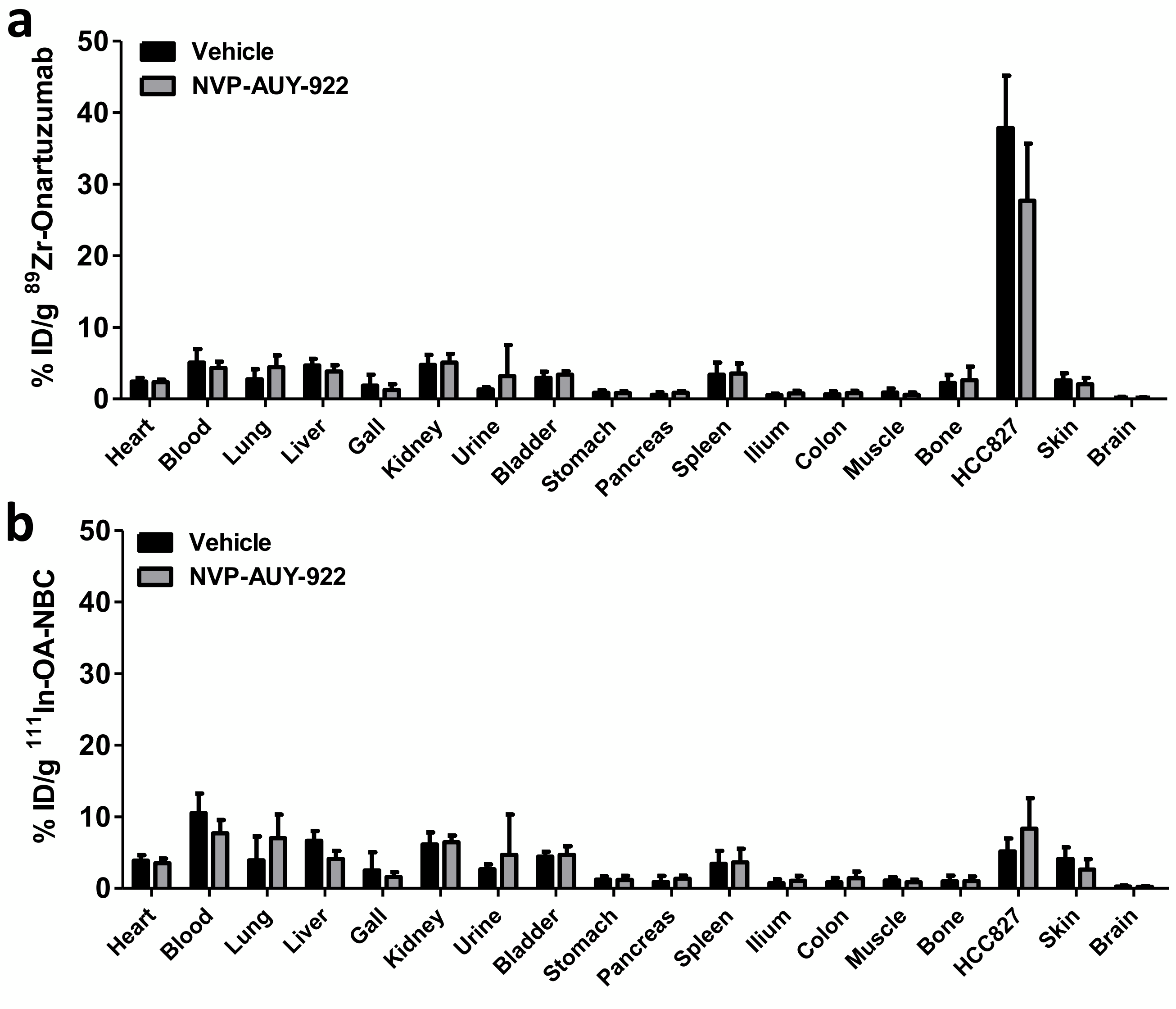

Supplement: Supplementary file 4 — a Ex vivo organ uptake of 89Zr-onartuzumab 6 days after injection in six vehicle-treated and seven NVP-AUY-922-treated (at a dose of 100 mg/kg) HCC827 xenograft-bearing mice. b Corresponding ex vivo organ uptake of 111In-OA-NBC 6 days after injection in HCC827 xenograft-bearing mice. Data are expressed as %ID/g ± SD (PDF 42 kb) [file 259_2017_3672_MOESM4_ESM.pdf]
