# Supplementary material for: 89Zr-Onartuzumab PET imaging of c-MET receptor dynamics
Source: Eur J Nucl Med Mol Imaging. 2017 Mar 19;44(8):1328–36. doi: 10.1007/s00259-017-3672-x (PMC5486818; doi:10.1007/s00259-017-3672-x)

Supplementary Fig. 5 Histologic grading of necrosis (H&E staining) of **(a)** HCC827 and HCC827ErlRes tumours and (b) vehicle and NVP-AUY-922 treated HCC827 tumours, with 0+ being 0-5% necrosis, 5-15% necrosis 1+, 15-25% necrosis 2+, 25-35% necrosis 3+ and over 35% necrosis as 4+.

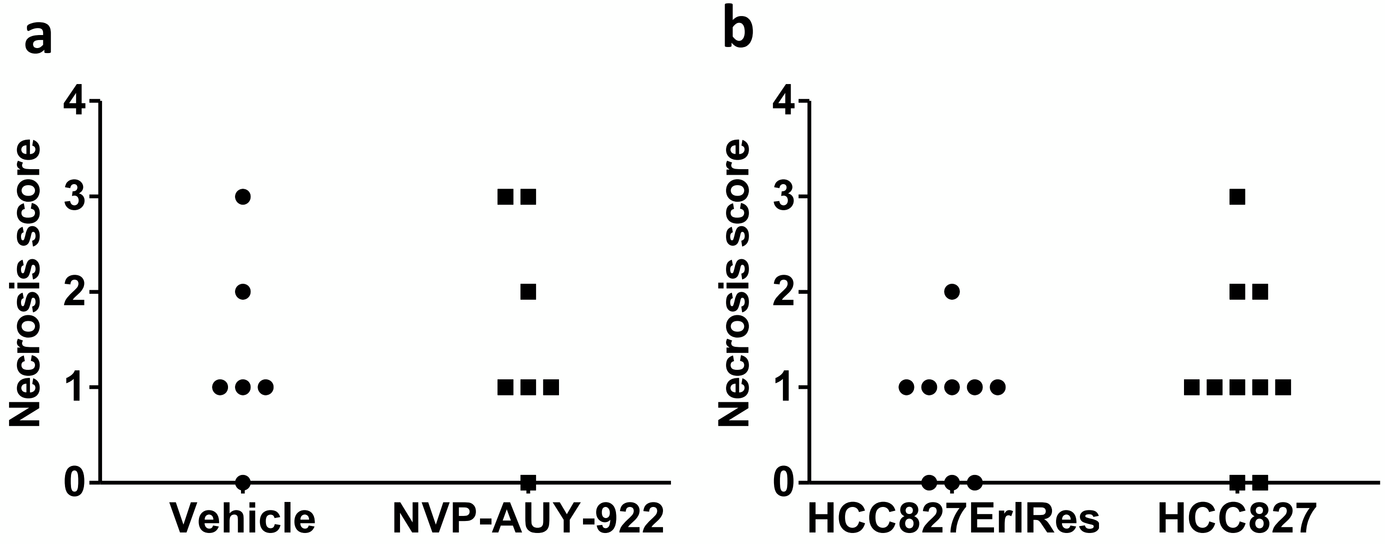

Supplement: Supplementary file 5 — Histological grading of necrosis (H&E staining) of (a) HCC827 and HCC827ErlRes tumours and (b) vehicle-treated and NVP-AUY-922-treated HCC827 tumours, where score 0+ represents 0–5% necrosis, 1+ 5–15% necrosis, 2+ 15–25% necrosis, 3+ 25–35% necrosis and 4+ >35% necrosis. (PDF 25 kb) [file 259_2017_3672_MOESM5_ESM.pdf]
