# Supplementary material for: 89Zr-Onartuzumab PET imaging of c-MET receptor dynamics
Source: Eur J Nucl Med Mol Imaging. 2017 Mar 19;44(8):1328–36. doi: 10.1007/s00259-017-3672-x (PMC5486818; doi:10.1007/s00259-017-3672-x)

Supplementary Fig. 6 Correlation of *ex vivo* ^{89}Zr-onartuzumab/^{89}Zr-OACD8 tumour uptake in % ID/g with *in vivo* PET tumour uptake in SUV_{mean}

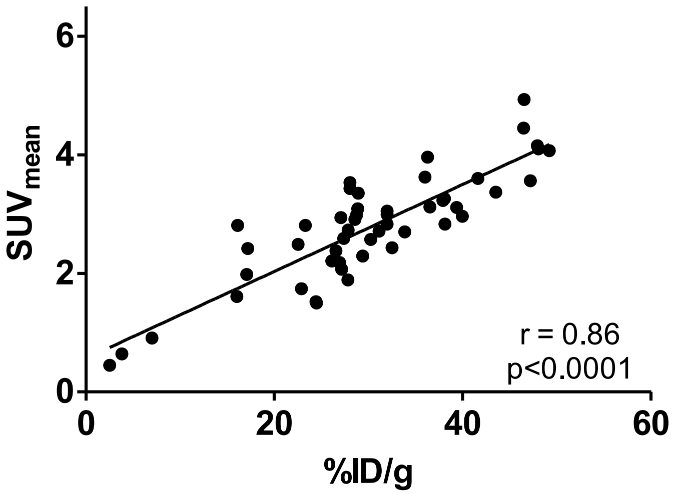

Supplement: Supplementary file 6 — Correlation between ex vivo 89Zr-onartuzumab/89Zr-OACD8 tumour uptake (%ID/g) and in vivo PET tumour uptake (SUVmean). (PDF 31 kb) [file 259_2017_3672_MOESM6_ESM.pdf]
